# Expression of a Single, Viral Oncoprotein in Skin Epithelium Is Sufficient to Recruit Lymphocytes

**DOI:** 10.1371/journal.pone.0057798

**Published:** 2013-02-26

**Authors:** Allison Choyce, Michelle Yong, Sharmal Narayan, Stephen R. Mattarollo, Amy Liem, Paul F. Lambert, Ian H. Frazer, Graham R. Leggatt

**Affiliations:** 1 The University of Queensland Diamantina Institute, University of Queensland, Princess Alexandra Hospital, Woolloongabba, Brisbane, Australia; 2 McArdle Laboratory for Cancer Research, University of Wisconsin School of Medicine and Public Health, Madison, Wisconsin, United States of America; University of Pittsburgh, United States of America

## Abstract

Established cancers are frequently associated with a lymphocytic infiltrate that fails to clear the tumour mass. In contrast, the importance of recruited lymphocytes during premalignancy is less well understood. In a mouse model of premalignant skin epithelium, transgenic mice that express the human papillomavirus type 16 (HPV16) E7 oncoprotein under a keratin 14 promoter (K14E7 mice) display epidermal hyperplasia and have a predominant infiltrate of lymphocytes consisting of both CD4 and CD8 T cells. Activated, but not naïve T cells, were shown to preferentially traffic to hyperplastic skin with an increased frequency of proliferative CD8+ T cells and CD4+ T cells expressing CCR6 within the tissue. Disruption of the interaction between E7 protein and retinoblastoma tumour suppressor protein (pRb) led to reduced epithelial hyperplasia and T cell infiltrate. Finally, while K14E7 donor skin grafts are readily accepted onto syngeneic, non-transgenic recipients, these same skin grafts lacking skin-resident lymphocytes were rejected. Our data suggests that expression of a single oncoprotein in the epidermis is sufficient for lymphocyte trafficking (including immunosuppressive lymphocytes) to premalignant skin.

## Introduction

The initiation of tumours not only requires genetic and epigenetic alterations to cell cycle control but also the generation of a supportive, extrinsic microenvironment. One important component of the microenvironment is inflammatory cells which can either promote or detract from tumour growth [1]. Triggers for the recruitment of inflammatory cells can include the activation of certain oncogenes such as Myc or Ras which attract mast cells and other innate immune cells through the release of soluble chemoattractants from the transformed cells [2], [3]. While reports of inflammation in cancer have generally focused on the activity of innate immune cells, the presence of infiltrating lymphocytes has also been recognised particularly in advanced solid tumours [4]. The arrival of lymphocytes at the tumour would be expected to associate with an anti-tumour immune response and clearance of the malignancy, although this is clearly not the case for many tumours. One view suggests that constant immune pressure drives the evolution of tumours towards immune escape and this has been elegantly described in the cancer immunoediting model [5]. Within this framework, cancer cells exist in an equilibrium state with the immune system for many years before eventually gaining attributes which negate the immune response. The escape of tumours from immune attack is now recognised as one of the hallmarks of cancer [6]. One strategy for obtaining the equilibrium state in the tumour microenvironment would be the recruitment of immunosuppressive lymphocyte subsets such as regulatory T cells. Consequently, a key question is whether premalignant lesions recruit a suppressive lymphocyte infiltrate to facilitate the equilibrium phase of tumour development.

We have an established mouse model of epithelial premalignancy in the skin whereby the human papillomavirus (HPV) E7 oncoprotein is expressed as a transgene within keratinocytes under the control of the keratin 14 transcriptional promoter. Expression of E7 protein in the skin keratinocytes results in epithelial hyperplasia that is stable and does not progress to invasive cancer up to at least 4 months of age. Previously, we have shown that E7-expressing skin grafts from these transgenic mice fail to be rejected when placed on syngeneic, non-transgenic mice that should see the E7 protein as foreign [7], [8]. Skin resident natural killer T (NKT) cells were shown to be one necessary component in the suppression of the immune response against the K14E7 skin grafts [9]. This study also suggested that NKT cells were part of a larger infiltrate of T cells within the K14E7 skin tissue. In the current study, we have determined that a lymphocytic infiltrate is preferentially recruited to premalignant skin via an interaction between the retinoblastoma tumor suppressor protein and the HPV-16 E7 oncoprotein. Our study also highlights that an immunosuppressive microenvironment is established prior to the formation of solid tumours and may assist with the persistence of premalignant lesions. Immunotherapeutic treatments for squamous cell carcinomas will need to overcome or disrupt the local tumour microenvironment in order to be successful.

## Materials and Methods

### Mice

K14E7 mice [10], in which the HPV16E7 protein is driven from a keratin 14 promoter on a C57BL/6 background, C57BL/6, B6.SJL-Ptprc (CD45.1) and RAG1^−/−^ mice were obtained from the Animal Resource Centre (Perth, Australia) and the Princess Alexandra Hospital Biological Research Facility (PAH-BRF) (Brisbane, Australia). K14E7×RAG1^−^/^−^ mice were bred at the PAH-BRF in addition to E7TCR-β transgenic mice on a C57BL/6 background that have been previously described [10]. K14SIY mice on the C57BL/6 background were a gift of Dr. Kristin Hogquist [11]. *Rb* ^ΔLXCXE^ (Rbmut) mice and K14E7x*Rb* ^ΔLXCXE^ mice on a mixed 129/FVB/C57 background have been previously described and were bred at the McArdle Laboratory Cancer Center Animal Care Facility [12].

### Ethics Statement

All animal procedures were performed under approved protocols assessed by the University of Queensland Animal Ethics Committee (UQDI/290/10/NHMRC/NIH (NF)). Skin grafting was performed under anaesthetic to minimise animal suffering.

### Reagents, flow cytometry and histology

E7 peptide (RAHYNIVTF)/H-2D^b^ pentamer was purchased from Proimmune (Oxford, UK). Fluorochrome-conjugated antibodies to murine CD8 (53–6.7), CD4 (RM4-4), CD3 (145-2C11), CD45.2(104), CD45.1 (A20), Ki67(B56), CCR6 (140706) and CCR4 (2G12) were purchased from R&D systems (Minneapolis, MN), eBioscience (San Diego, CA), BD Biosciences (San Jose, CA) and Biolegend (San Diego, CA). For flow cytometry, isolated single cell suspensions from skin were preincubated with Fc block (anti-mouse CD16/32; BD Bioscience) and, in some experiments, Live/Dead Cell Near Infrared Dead cell stain (Invitrogen Product No. L10119, Life Technologies, Mulgrave, Australia), for 10–30 mins on ice before incubation with fluorochrome-conjugated antibodies diluted in PBS +2% heat-inactivated FCS for 30 mins-1 hr. Stained cells were fixed using a 10% formalin solution before analysis on a FACsCalibur or FACsCanto flow cytometer (BD Biosciences). Forward and side scatter properties were used to define a lymphocyte gate which was applied to all samples in our dataset. In some experiments, flow-count fluorospheres (Beckman Coulter) were included to enable an absolute cell count to be established. Intracellular detection of Ki67 was performed, after staining of surface markers, using a BD Cytofix/Cytoperm kit according to manufacturer's instructions (BD Biosciences). For histological sections, ear skin tissue was placed in a 10% formalin solution, embedded in paraffin and then sections cut using a microtome. Mounted sections were stained with haematoxylin and eosin before sections were photographed under light microscopy.

### Isolation of a single cell suspension from full thickness ear skin

The isolation of skin cells from mouse ears has been previously described [9]. Briefly, mouse ears were mechanically divided into dorsal and ventral halves before the tissue was teased apart using forceps and incubated in 20 U/mL Dnase I and 0.1 U/mL of collagenase or 0.1 U/mL Collagenase +0.8 U/mL Dispase in PBS (Roche, Berlin, Germany). Following digestion, the cell preparation and remaining fragments were washed through a 70 µM cell strainer with PBS +2% FCS to create a single cell suspension.

### Skin Grafting

Recipient mice were grafted on the flank with ear skin grafts as previously described by our group [9], [13]. Briefly, both ventral and dorsal ear skin grafts (approx. 1 cm^2^) were placed on the thoracic flank region of anaesthetised, recipient mice and secured using antibiotic-permeated gauze (Bactigras; Smith and Nephew, London, U.K.) in addition to bandaging with micropore tape and Flex-wrap (Lyppard, Queensland, Australia). All bandages were removed after 7 days and grafts were monitored for signs of rejection. Grafts were recorded as rejected after an 80% reduction in the graft size.

### Activation of T cells and adoptive transfer

Spleens were removed from mice and a single cell suspension prepared by mechanical disruption through a 70 µM cell strainer. Red blood cells were removed by ACK lysis buffer. To non-specifically activate T cells, splenocytes were incubated with 25 ng/mL PMA/1 µg/mL Ionomycin for 3 days at 37°C, before washing the cells twice with PBS, counting live cells with Trypan blue and transferring intravenously into mice at 7.5×10^6^–1×10^7^ live cells/mouse. Live cells were estimated to be 60–65% of the culture at the end of the three day stimulation. In some experiments, splenocytes were prepared as a single cell suspension (naive T cell transfer) and directly transferred into recipient mice.

For analysis of E7/H-2D^b^ pentamer binding cells, K14E7 or C57BL/6 mice (both CD45.2) received 1×10^7^ splenocytes from CD45.1^+^ E7TCRVβ transgenic mice. At different time points, the spleen or ear skin cells were harvested and stained with CD45.1 and CD8 antibodies and E7/H-2D^b^ pentamer (Proimmune, Oxford, UK). Data was analysed as the percentage of CD45.1^+^CD8^+^ with or without E7/H-2D^b^ pentamer of total lymphocyte-sized cells (based on forward and side scatter properties) in the spleen or skin.

### Statistics

Skin graft survival was plotted using Kaplin-Meier plots with a Log rank test used to determine significant differences in the curves. All other data was assessed using a two sided, non-parametric Mann-Whitney test with P<0.05 considered to be statistically significant. All graphs and statistical tests were prepared using Prism software (Graphpad Software, La Jolla, CA).

## Results

### Adoptively transferred T cells traffic to K14E7 mouse skin

**Figure 1:**
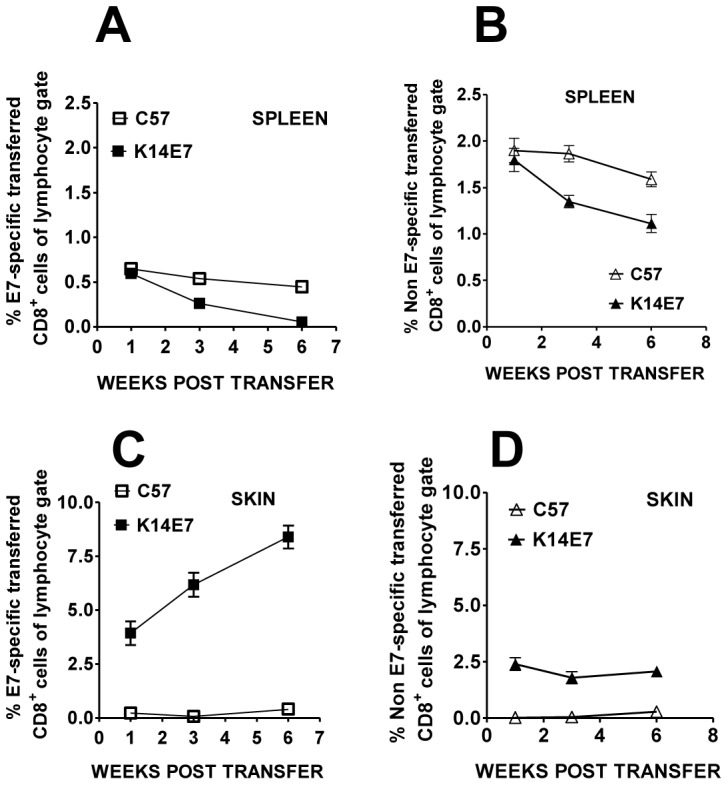
Transferred E7TCR-β transgenic CD8 T cells accumulate in K14E7 transgenic skin. E7TCR-β × CD45.1 transgenic splenocytes (1×10^7^) were transferred intravenously to either K14E7 (CD45.2) or control C57 (C57BL/6) mice. The percentage of CD45.1^+^ CD8^+^ T cells that did (A and C) or did not (B and D) bind E7 peptide/MHC pentamer as a fraction of the total lymphocytes (lymphocyte-sized gate based on forward versus side scatter properties) was assessed in the spleen (A and B) or ear skin (C and D) of K14E7 and wild type mice at varying time points post transfer. Error bars on some plots are too small to be seen. The data represent the mean and standard error of 4 K14E7 and 4 C57BL/6 mice total at each time point derived from 2 independent experiments.

In an earlier study, our group had shown that T cell function in the spleen of K14E7 transgenic mice was suppressed at least partly by CD4^+^CD25^+^ cells [10]. It was also possible that a component of this apparent suppression was the redistribution of E7-specific lymphocytes to other organs such as the skin where antigen is expressed. To address the tissue distribution of E7-specific lymphocytes in K14E7 mice, we tracked the fate of CD45.1^+^CD8^+^ E7TCR-β chain transgenic cells transferred into CD45.2^+^ K14E7 mice. Given that the TCR beta chain is only one half of a dimeric receptor also containing a TCR alpha chain, CD8 T cells from these transgenic mice are enriched in E7-specific T cells but do harbour CD8^+^ cells (and CD4^+^ T cells) with unrelated specificities thus allowing the tracking of multiple lymphocyte populations. Splenocytes, from E7TCR-β transgenic mice, were transferred into K14E7 or control mice (C57) and the localisation of E7-specific versus non E7-specific transferred (CD45.1^+^) CD8^+^cells within spleen or skin tissue was monitored using E7 peptide/H-2D^b^ pentamers. Although a similar fraction of E7-specific (Fig. 1A) and non-specific T cells (Fig. 1B) was recovered from the spleen of K14E7 and C57 control mice 1 week post-transfer, the fraction of input, CD45.1^+^CD8^+^ T cells slowly declined in this tissue out to 6 weeks. In contrast, E7-specific CD45.1^+^ T cells became enriched over time within the skin of these same K14E7 mice while no significant time dependent enrichment was seen in non-transgenic animals (Fig. 1C). The nontransgenic mice had much lower fractions of the input CD45.1^+^, E7-specific T cells in their skin compared to the K14E7 transgenic mice over the whole time course. The fraction of CD45.1^+^ non-E7 specific CD8 T cells was also higher in the skin of K14E7 mice compared to non-transgenic (Fig. 1D). This suggested that the K14E7 skin environment was generally permissive to T cell trafficking although antigen-specificity of the T cells assisted in accumulation over time. Consequently, we looked to characterise the properties of T cells which allowed trafficking to precancerous skin.

**Figure 2:**
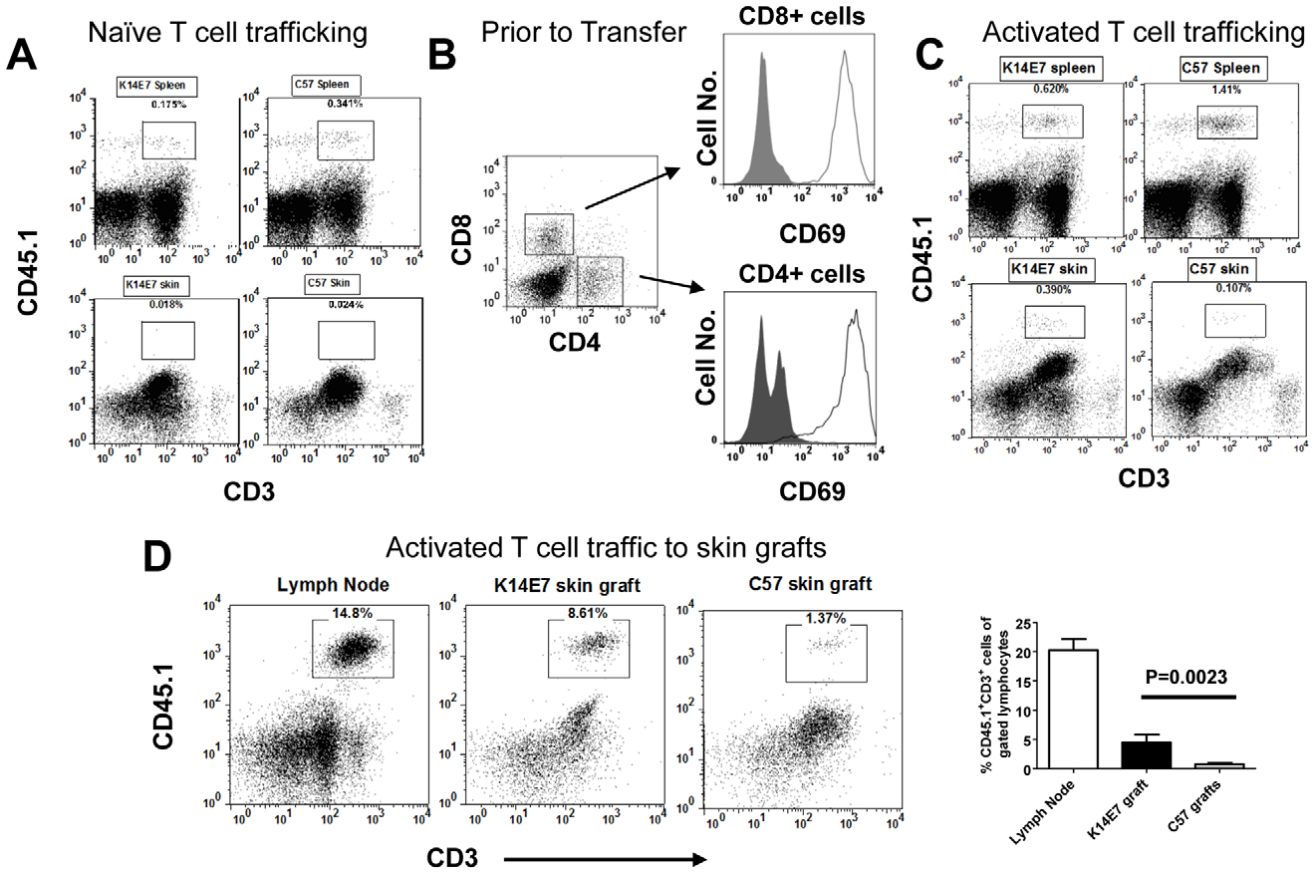
Activated but not naïve T cells accumulate preferentially in K14E7 ear skin. (A) Naïve splenocytes (1×10^7^) derived from C57.SJL (CD45.1) mice were intravenously transferred into K14E7 or C57BL/6 mice (CD45.2). After 7 days, the ear skin (bottom panels) and spleens (top panels) from both sets of recipient mice was harvested and analysed by flow cytometry for CD45.1 and CD3 staining. Plots are representative of 4–6 individual mice over two independent experiments. (B) Splenocytes from B6.SJL (CD45.1) mice were activated with PMA/Ionomycin for 3 days before staining for CD4, CD8 and CD69 expression immediately prior to in vivo transfer. Histograms were gated on either CD4 or CD8 T cell populations with shaded plots representing isotype control staining and open plots representing CD69 expression. (C) Activated splenocytes generated in (B) were transferred intravenously (1×10^7^ cells) into K14E7 or C57BL/6 mice. Both the spleen (top panels) and ear skin (bottom panels) was harvested from the recipient mice and CD45.1 and CD3 expression was assessed by flow cytometry. Plots are representative of 6 individual mice over two independent experiments. (D) K14E7 and C57BL/6 ear skin was grafted onto the same flank of a RAG1^−/−^ mice and allowed to heal. B6.SJL splenocytes were activated for 3 days in vitro with PMA/Ionomycin before transfer (1×10^7^) into skin grafted, recipient RAG1^−/−^ mice. After 7 days, skin grafts were removed and analysed for transferred T cells using flow cytometry. Representative plots are shown in addition to a summary graph of the dataset (lower panel) showing the means + SEM.

To ascertain if skin trafficking required activation of T cells, naïve and in vitro activated (PMA+Ionomycin) splenocytes from non-transgenic, CD45.1^+^ mice were transferred into K14E7 and control mice bearing CD45.2. Transferred, naive T cells were detected in the spleen of recipient mice but were infrequent in either K14E7 or control skin (Fig. 2A). In contrast, PMA/Ionomycin activated T cells, expressing the early activation marker, CD69, at the time of transfer (Fig. 2B), showed preferential trafficking to K14E7 skin relative to control C57BL/6 skin (Fig. 2C, lower panels). Activated T cells were also detected in the spleen of K14E7 and control mice (Fig. 2C). To confirm that trafficking was preferential for K14E7 skin and not a product of the K14E7 transgenic environment, we compared the accumulation of activated T cells transferred into RAG1^−/−^ mice that were grafted with both a healed C57BL/6 and a healed K14E7 skin graft. Over the period of 10 days, activated T cells were seen to accumulate in K14E7 skin grafts rather than the neighbouring C57BL/6 graft suggesting preferential attraction to the transgenic skin in an otherwise non-transgenic mouse (Fig. 2D).

### Endogenous, skin resident T cells are increased in K14E7 mice

**Figure 3:**
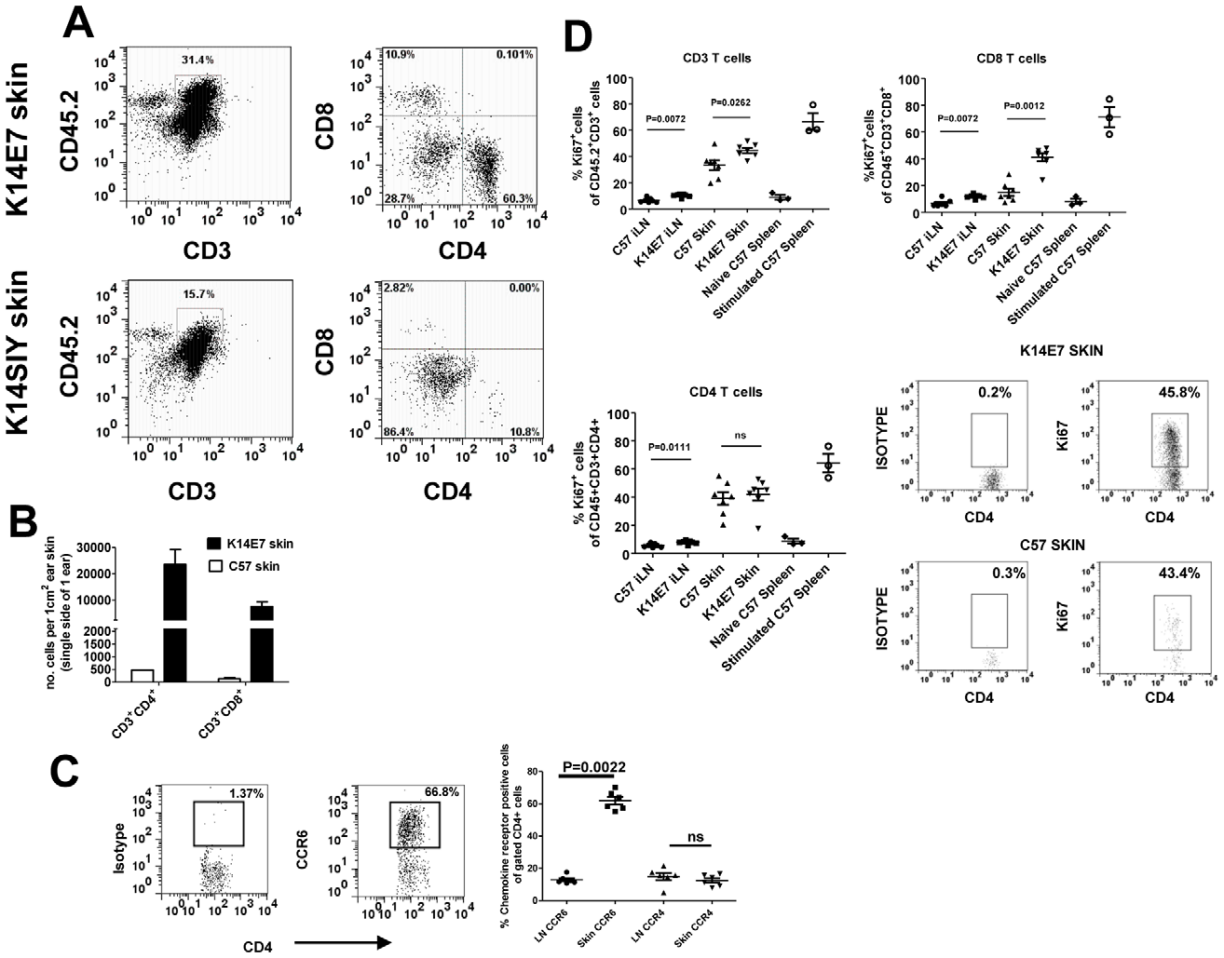
Endogenous T cells accumulate in K14E7 skin with an enrichment for CCR6-expressing CD4 T cells. (A) Ear skin tissue was taken from K14E7 mice in addition to control mice expressing the SIY epitope under the K14 promoter. CD4 and CD8 expression was measured on gated CD45^+^CD3^+^ cells. Data is representative of at least 4 mice/group (B) The numbers of CD4^+^ and CD8^+^ T cells in K14E7 skin or control C57 skin were enumerated per square centimetre of ear skin using flow count beads in flow cytometry. Data represents pooled mice from at least two independent experiments. (C) Gated CD3^+^ CD4^+^ T cells from the lymph node or skin of K14E7 mice were analysed for expression of the chemokine receptors, CCR6 and CCR4. The left hand panels show representative plots of isotype and CCR6 antibody staining for CD4^+^ T cells while the graph summarises chemokine receptor staining representative of 6 mice/group from 3 independent experiments. (D) Both K14E7 mice and C57 mice were analysed for the proliferative marker, Ki67, using intracellular staining of lymphocytes derived from the skin and inguinal lymph nodes (iLN). Naive C57 spleen cells (negative control) or C57 spleen cells treated for 3 days with PMA/Ionomycin (positive control) were also analysed. The K14E7 and C57 data represent 7 mice/group derived from 3 independent experiments while controls are spleen cells from a single mouse in 3 independent experiments. The lower right hand panels are representative plots showing isotype and Ki67 staining in K14E7 or C57 mouse skin.

Having demonstrated the trafficking of transferred, activated T cells to K14E7 skin, we hypothesized that endogenous T cells should also be increased in the skin of K14E7 mice relative to littermate controls. Full thickness, ear skin (where E7 expression is greatest) was removed from adult K14E7 mice along with control K14 transgenic mice (K14SIY) and C57BL/6 mice for analysis of lymphocyte infiltrate. In this experiment, collagenase alone was used to isolate skin cells as dispase was shown to affect the staining intensity of anti-CD4 and CD8 antibodies. Under these conditions, CD45^−^ cells had higher background staining then seen in other figures but the staining intensity of CD45^+^ (or CD3^+^) cells was not affected. The E7 transgenic mice had an increased proportion of CD3^+^CD45^+^ cells including a larger percentage of CD4^+^ and CD8^+^ T cells relative to control mice although the ratio of CD4^+^ to CD8^+^ cells was similar (Fig. 3A). CD4^+^ T cells were more frequent than CD8 T cells in the skin of all the mice (Fig. 3A) with approximately half of all CD4 T cells in either K14E7 or control skin expressing the regulatory T cell marker, FoxP3 (Leggatt et al. -data not shown). These differences were also reflected in absolute cell counts from the skin where CD4 and CD8 T cells were more numerous in K14E7 skin relative to control mice (Fig. 3B). The skin cell count for CD8 T cells per square centimetre in control C57BL/6 mice was consistent with previous estimates in the literature [14]. This suggested that the accumulation of lymphocytes was due to E7 expression in the skin.

Chemokine receptors involved in conventional homing of T cells to the skin include CCR4, CCR10 and CCR6 [15]. We found an enriched proportion of CCR6^+^ CD4^+^ T cells in the skin of K14E7 mice relative to the skin draining lymph node (Fig. 3C). In contrast, the proportion of CD4^+^ T cells expressing CCR4 was not different between the skin and lymph node (Fig. 3C). Skin CD8^+^ T cells showed a similar but more modest enrichment of CCR6^+^ cells (as a percentage of CD8^+^ T cells) in the skin (26%+/−2.2; mean +/− SEM(n = 6)) relative to lymph nodes (7.7%+/−0.63; mean +/− SEM (n = 6)). The fraction of CD8^+^ cells expressing CCR4 in skin or lymph node was less than 5%.

Accumulation of lymphocytes in K14E7 skin tissue represents increased T cell trafficking but equally could represent increased proliferation of lymphocytes within the tissue. To address differences in proliferative capacity between lymphocytes in K14E7 skin and control skin, we assessed the expression of Ki67, a known marker of cells undergoing proliferation [16]. Interestingly, almost half the CD3^+^ T cells in either transgenic or control skin expressed the Ki67 marker in contrast to the lymph nodes where less than 15% of the T cells were positive (Fig. 3D). K14E7 mice had a statistically significant increase in the fraction of proliferative cells relative to control mice in both the lymph nodes and skin. When separated into CD4^+^ and CD8^+^ T cells, the key difference in proliferating cells was seen for CD8^+^ T cells where almost double the fraction of Ki67^+^ cells was seen in K14E7 mice relative to C57 control mice. Overall, this data suggested that small differences in proliferative capacity of the skin T cells between K14E7 and C57 mice might contribute to the T cell enrichment in the skin.

### Skin resident lymphocytes in K14E7 mice functionally impair the immune response

**Figure 4:**
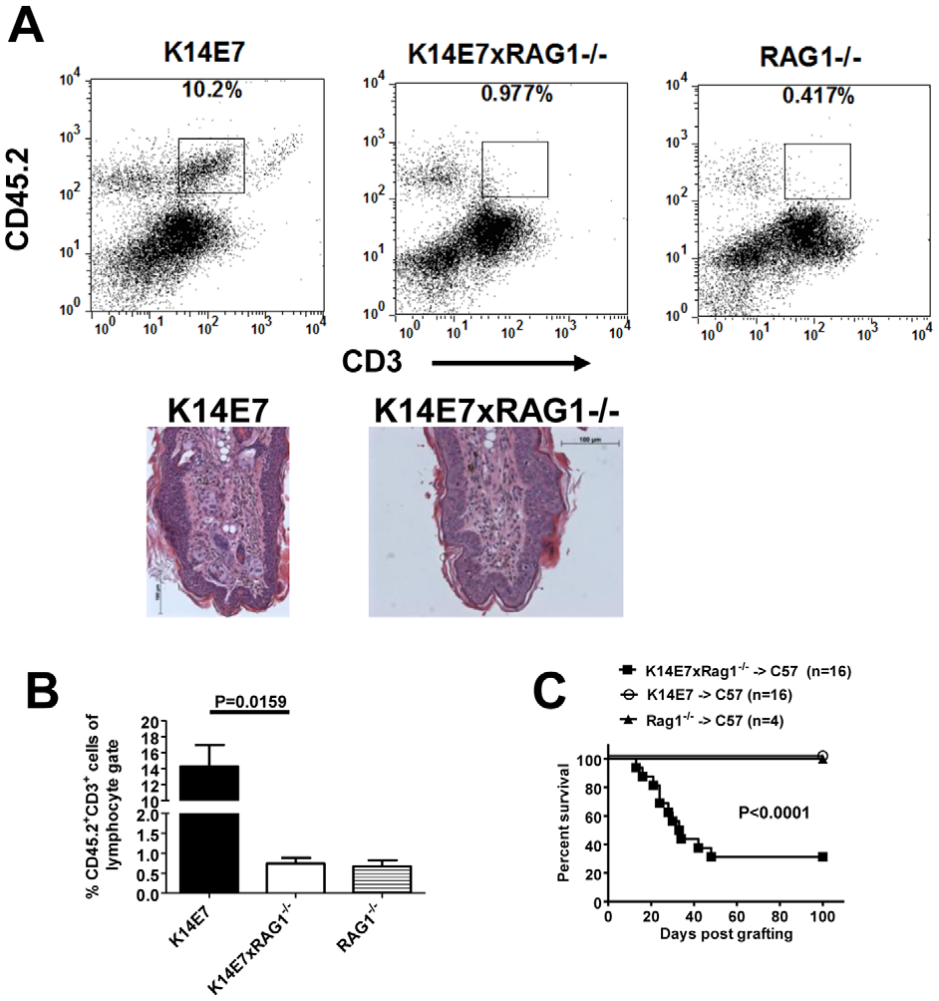
Lymphocyte-deficient K14E7 ear skin is more susceptible to immune-mediated rejection. (A) Representative flow cytometry plots of CD45 and CD3 staining in ear skin derived from K14E7, K14E7×RAG1^−/−^ and RAG1^−/−^ mice. Haematoxylin and eosin staining of ear skin tissue sections taken from K14E7 and K14E7×RAG1^−/−^ mice are shown. (B) A graph summarizing the proportion of CD45.2^+^CD3^+^ cells seen in each of the representative plots from (A). Data represents 3–5 individual mice/group from 2 independent experiments (C) Ear skin from K14E7, K14E7×RAG1^−/−^ and RAG1 mice was grafted onto the flank of C57BL/6 mice and the percent of mice with surviving grafts was plotted against time post grafting. The K14E7 and K14E7×RAG1^−/−^ grafting groups contained 16 mice/group while the RAG1^−/−^ group contained 4 mice from at least 2 independent experiments.

To test whether the presence of lymphocytes in the K14E7 skin had an impact on immune responses directed against E7, we crossed K14E7 mice with RAG1^−/−^ mice which are T and B cell deficient due to a lack of the recombinase enzyme involved in lymphocyte receptor rearrangement. Ear skin was examined in the K14E7×RAG1^−/−^ to confirm a lack of resident T cells. The CD45^+^ cells showed a clear population of CD3^+^ T cells in K14E7 mice which was absent in K14E7×RAG1^−/−^ mice or RAG1^−/−^ mice (Fig. 4A+B). These plots also highlight that an apparent CD45^+^ CD3^+^ dim population seen in earlier figures is unlikely to represent a true population of T cells. In addition, this dim population does not stain for CD4 or CD8 in flow cytometry (unpublished data). It was also noted that epithelial hyperplasia, observed in histological sections, was unaffected by the presence or absence of lymphocytes (Fig. 4A lower panels). Importantly, when K14E7 and K14E7×RAG1^−/−^ grafts were placed on recipient, immunocompetent C57BL/6 mice, skin graft rejection was seen in the majority of K14E7×RAG1^−/−^ mice lacking donor lymphocytes (Fig. 4C). In contrast, skin grafts from RAG1^−/−^ mice or K14E7 mice harbouring lymphocyte infiltrates were not rejected. K14E7×RAG1^−/−^ skin grafts onto RAG1^−/−^ mice were not rejected (unpublished data) suggesting that wound healing was not affected in these skin grafts and that graft rejection required recipient lymphocytes. We cannot rule out that other genetic abnormalities (beyond loss of lymphocytes) exist in the K14E7×RAG1^−/−^ mice and might contribute to skin graft rejection. However, the rejection of K14E7×RAG1^−/−^ grafts is entirely consistent with the immunosuppressive role of skin-resident NKT cells that we demonstrated in earlier studies [9] and does not rule out a suppressive role for other skin resident, donor lymphocytes.

### Retinoblastoma (Rb) protein is important for K14E7 driven epithelial hyperplasia and the development of lymphocytic infiltrate

**Figure 5:**
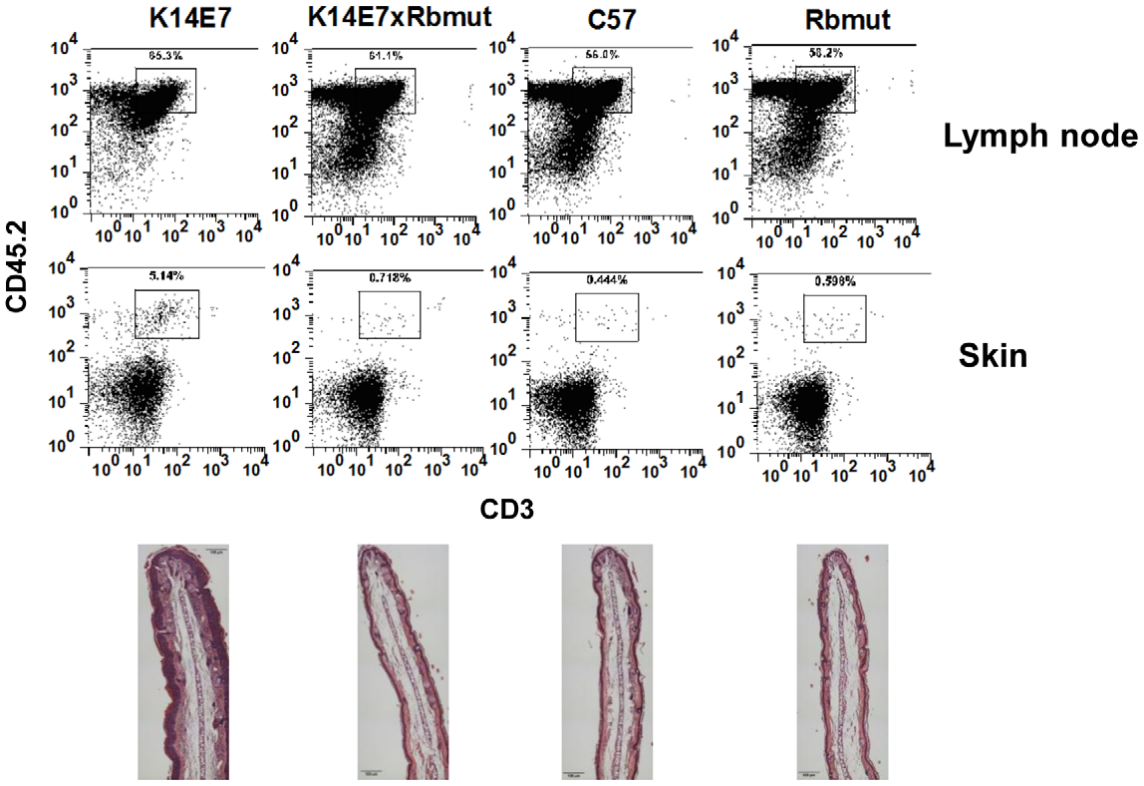
Reduced T cell infiltrate in K14E7 mice containing mutant Rb. Representative FACs plots are shown for ear skin from K14E7, K14E7xRb mutant, C57BL/6 and Rb mutant mice stained with CD45 and CD3 (top panels). The data for the K14E7xRb mutant was representative of 4/5 mice tested with the outlying mouse having an unusually high T cell fraction in the skin of 20%. The lower panels are representative haematoxylin and eosin staining of ear skin from each of the corresponding mice listed in the top panels.

The HPVE7 oncoprotein mediates its affects on proliferation and cell cycle via binding and inactivation of the tumour suppressor gene, retinoblastoma (Rb) protein [17]. To determine the role of this interaction in the recruitment of T cells to the skin, we utilized a knock-in mouse engineered to express a mutant Rb that does not bind to the E7 protein [12]. When the K14E7 mice were crossed with mutant Rb mice, epithelial hyperplasia in the skin was lost (Fig. 5, lower panels) as we had shown previously [12]. Ear skin from these mice also showed a decreased infiltrate of CD45^+^CD3^+^ T cells which now approximated the proportions found in control animals (Fig. 5). It was noted that the proportion of CD45^+^CD3^+^ cells in the skin draining lymph nodes of these same mice were not significantly different suggesting a selective decrease in the ear skin (Fig. 5). This data suggested that the ability of E7 to induce lymphocyte infiltrate correlates with its induction of epithelial hyperplasia and both phenotypes are dependent upon E7's inactivation of the tumour suppressor pRb.

## Discussion

While tracking the fate of transferred E7-specific CD8 T cells in K14E7 mice we have identified a selective trafficking of polyclonal, activated CD4 and CD8 T cell populations to premalignant, hyperplastic skin. In the presence of a conventional inflammatory signal within the skin such as pathogen infection, trafficking of the lymphocytes would not be surprising and serves the role of defending the skin barrier [18]. In contrast, the role of cell transformation or cell stress in generating inflammatory signals and leukocyte trafficking is less understood and currently under intense investigation [19], [20]. Few reports have associated a single oncoprotein such as HPVE7, in the absence of viral infection and other HPV proteins, with a dominant lymphocytic infiltrate into the skin. Other studies using K14-HPV16 transgenic mice (expressing multiple HPV genes including E6 and E7) support the presence of mild lymphocytic infiltrates which increased in older mice with dysplastic skin and was promoted by the presence of *Staphylococcus* [21], [22]. In these studies, a function for accumulated lymphocytes in the skin was not determined. The presence of lymphocytes in K14E7 skin would also be consistent with lymphocyte infiltrates seen in human cervical cancer and precursor lesions [23], [24].

Other activated oncogenes (*Myc, Ras*) have generally been reported to promote an innate immune infiltrate, cytokine secretion or angiogenesis [2], [3], [25], [26]. It is likely that recruitment of innate inflammatory cells in these models will eventually lead to the attraction of lymphocytes although this was not reported. Each oncoprotein invokes different signalling pathways within the epithelial cell and this will likely influence the pattern of secreted cytokines/chemokines and subsequent immune infiltrate. Therefore, making broader predictions about cell trafficking to premalignant lesions is complicated by the heterogeneity in tumour initiating events. In addition, the type of tissue in which the tumour arises may play a role in chemoattraction [27].

Our data support a model in which HPV E7 protein binding to pRb protein initiates signalling pathways that drive a sterile, chronic inflammatory environment within the skin evidenced by a lymphocytic infiltrate. Hyperproliferation and a resulting excess of secreted chemoattractants from the skin epithelium would represent one signal which might drive the accumulation of lymphocytes in the skin. To this end, the presence of an enriched population of CCR6^+^ CD4 T cells within the skin suggests that chemoattraction may play a role for this subset of lymphocytes. It is also consistent with our detection of increased levels of CCL20 by microarray in K14E7 whole skin extracts (Leggatt et. al. – data not shown). One study in colorectal cancer has demonstrated that tumour associated macrophages secrete CCL20 and are responsible for the recruitment of CCR6^+^ regulatory T cells which enhance tumour development [28]. A number of chemokine/chemokine receptor antagonists are being developed for tumour immunotherapy and it remains to be seen whether blocking of CCR6/CCL20 would impact on immunosuppression in squamous cell cancers [29], [30].

The extent to which proliferating epithelium, in the absence of E7 expression, controls lymphocyte accumulation in the skin can only be dissected using non-E7 models of hyperproliferative epithelium. Dissociation of E7 from its binding partner, Rb protein, in our system lead to a decrease in both hyperplasia and lymphocyte infiltrate. While these events are associated, we cannot rule out that a loss of Rb dependent functions unrelated to proliferation caused the lymphocyte reduction. Any possible link will be important to establish in future studies as it predicts that anti-proliferative cancer drugs will alter the immune microenvironment of skin tumours and potentially favour tumour clearance.

Accumulation of lymphocytes in premalignant tissue may also be caused by extensive proliferation of small numbers of skin infiltrating cells. Our data suggested that a large fraction of skin resident T cells in both control mice and K14E7 mice expressed the Ki67 proliferative marker relative to the low expression in the lymph node. The trigger for CD4^+^ and CD8^+^ T cell proliferation in the skin is not known although we cannot discount that proliferative signals were first acquired in the lymph node prior to skin trafficking. The constitutive proliferation of skin resident CD4^+^ cells rather than CD8^+^ cells is consistent with recent findings of CD4^+^ Treg proliferation in normal human skin [31]. The increased proportion of proliferative lymphocytes in K14E7 skin along with activated T cell recruitment suggests that the accumulation of lymphocytes in our model is multifactorial. The microenvironmental triggers for the difference in the fraction of proliferating CD8^+^ T cells between K14E7 mice and C57BL/6 mice remains to be explored. The proliferation of E7-specific CD8^+^ T cells may play some role based on the increasing numbers of skin CD8 T cells seen in our transfer system (Fig. 1).

T cells with the potential to be immunosuppressive are attracted to skin and include both NKT cells and FoxP3^+^ Treg cells. While we have demonstrated that NKT cells can be suppressive in K14E7 skin through production of IFN-g [9], skin-resident regulatory T cells appear to be redundant or unnecessary in the rejection of K14E7 ear skin grafts [32]. This contrasts with the important role of circulating Treg in suppressing CD8 T cell function in K14E7 lymphoid organs [10] and suggests tissue-specific roles for this T cell subset. The ability of other recruited T cell subsets in the K14E7 skin to be suppressive remains to be tested.

In this study, we have demonstrated that the earliest events in tumour development can involve recruitment of activated T cells which may suppress anti-tumour immunity. In earlier studies, we have identified NKT cells as one such suppressive population in the K14E7 skin but this does not exclude a role for other recruited lymphocytes or resident γδ T cells [9]. The recruitment of bulk lymphocytes, lacking significant numbers of anti-tumour effector cells, during premalignancy might represent a tumour strategy to limit the immune response and thus establish an equilibrium with the immune system. Depletion of tumour associated lymphocytes (using drug treatment or irradiation) prior to the delivery of immunotherapy may therefore represent a viable treatment option for early stage squamous cell carcinomas.
